# Impact of multifocal soft contact lenses on the shape discrimination threshold under glare in myopic children

**DOI:** 10.3389/fmed.2025.1635583

**Published:** 2025-08-29

**Authors:** Cuiting Huang, Xiuting Li, Jufen Liu, Jingjing Wu, Yuqing Wang, Lingli Lin, Jinfeng Zhang, Yanrong Chen, Zhaode Zhang, Li Li

**Affiliations:** ^1^Ophthalmology Department, Ningde Municipal Hospital of Ningde Normal University, Ningde, China; ^2^Ophthalmology Department, Children's Hospital of Fudan University at Xiamen, Xiamen, China; ^3^Ophthalmology Department, Shangyu People's Hospital of Shaoxing City, Shaoxing, China; ^4^Ophthalmology Department, Fuzhou University Affiliated Provincial Hospital, Fuzhou, China

**Keywords:** multifocal soft contact lenses, shape discrimination threshold, glare, myopic children, spectacles

## Abstract

**Background:**

Multifocal soft contact lenses (MFSCLs) are widely used to control myopia progression in children. However, their optical design may affect visual quality, especially under glare conditions, potentially influencing their daily visual performance.

**Purpose:**

To evaluate the impact of MFSCLs on shape discrimination threshold (SDT) under glare in myopic children by comparing changes in SDT under glare and non-glare conditions, with both MFSCLs and single vision spectacles.

**Methods:**

Thirty-seven myopic children (37 eyes) were enrolled and fitted with both MFSCLs and single vision spectacles for binocular myopia correction. Assessments included uncorrected and corrected visual acuity, corneal curvature, pupil diameter (PD) in a dark environment, and SDT with glare (SDTon) and without glare (SDToff). Measurements of the right eye were analyzed. SDTs were compared between lens types and lighting conditions, and correlations with ocular parameters were evaluated.

**Results:**

All participants achieved optimal MFSCL fit. No significant difference in SDTon or SDToff was observed between MFSCLs and spectacles (*P* > 0.05). However, SDTs were significantly elevated under glare compared to non-glare conditions (*P* < 0.001). In the MFSCL group, SDTon was significantly correlated with PD in the dark (*r* = 0.336, *p* = 0.042), as was the glare-induced SDT change (SDTdiff; *r* = 0.354, *p* = 0.032). In the spectacle group, SDTdiff was significantly associated with spherical equivalent refractive error (*r* = 0.435, *p* = 0.007). No serious adverse events occurred, and mild corneal staining resolved with appropriate care.

**Conclusion:**

MFSCLs did not significantly worsen SDTs under glare in myopic children compared to spectacles, suggesting they do not exacerbate disability under glare. These findings support the continued clinical use of MFSCLs for myopia management without compromising visual performance under glaring conditions.

## 1 Introduction

Myopia is a significant global health issue and has emerged as one of the leading causes of visual impairment and preventable blindness. In recent years, the global prevalence of myopia has increased substantially, with estimates suggesting that more than 4.7 billion people will be affected by myopia by 2050, including 938 million with high myopia (1, 2). Excessive axial elongation in patients with high myopia significantly increases the risk of sight-threatening complications, including macular degeneration and retinal detachment (3). Studies have demonstrated that various interventions, such as outdoor activities (4), atropine eye drops (5), bifocal spectacles (6), peripheral defocus spectacles (7), orthokeratology lenses (8–10), and multifocal soft contact lenses (MFSCLs) (11, 12), can effectively slow the progression of myopia in children. MFSCLs have been shown to slow myopia progression by ~0.21 D/year and limit axial elongation by ~0.11 mm/year (11, 12), making them one of the effective methods for controlling myopia progression in children. MFSCLs feature either an aspheric progressive design or an alternating concentric circle design, both of which slow axial elongation by generating a myopic defocus image (13).

MFSCLs, made from hydrophilic materials with adequate oxygen permeability, offer comfort and flexibility for wearers. Compared to orthokeratology lenses, they are easier to fit and are not limited by the patients' age. Consequently, MFSCLs have gained increasing popularity as an effective intervention for slowing myopia progression in children. However, wearing MFSCLs may lead to decreased visual quality and symptoms such as glare and halos, particularly in low-light conditions at night (14). Some wearers still report decreased visual quality even when their visual acuity is corrected to 0.00 logMAR or higher (15). This decreased quality is often manifested as subjective discomfort symptoms, including glare, halos, and double images (16, 17). Glare is a significant visual impairment that can negatively affect visual health and quality of life. It can be classified into three categories based on its mechanisms: disability glare, discomfort glare, and light-adapted glare (18–20).

Numerous studies emphasize the critical impact of glare on visual quality (21, 22). Maintaining good visual quality is essential for enhancing compliance with MFSCLs in myopic children, thereby supporting effective myopia control. It is crucial to investigate the impact of MFSCLs on myopic children with glare. Subjective visual quality scales, frequently employed in glare assessment, are significantly influenced by various factors (23). The accuracy of stray light photometric measurements can vary significantly between subjects, which currently limits their clinical application and large-scale use (24). The Oculus C-Quant is primarily used for the clinical evaluation of glare caused by corneal astigmatism, cataracts, and other ocular conditions (25).

In a study on glare by Aslam et al. (26), it was found that disability glare primarily results from a reduction in retinal imaging contrast due to intraocular stray light. Glare is typically assessed by measuring contrast sensitivity. However, studies by Plainis and Murray (27), Hiraoka et al. (28), and others have shown that the effects of glare on visual function may involve mechanisms beyond a mere reduction in visual contrast. In real-world settings, the human visual system relies on higher-order processing to detect subtle differences in shape and form. Shape discrimination tasks, which require such nuanced perception, have thus emerged as a promising method to evaluate visual performance under glare conditions.

In this study, we used a shape discrimination-based glare evaluation paradigm to examine the effects of MFSCLs on glare-related visual function. By measuring SDTs under glare and non-glare conditions in children wearing MFSCLs and spectacles, we aimed to gain deeper insights into the functional impact of MFSCLs and to inform clinical decision-making in pediatric myopia management.

## 2 Materials and methods

### 2.1 Study population

This study enrolled 37 pediatric patients with myopia (37 right eyes) who visited Ningde City Hospital, Affiliated to Ningde Normal University, between June 2020 and June 2022. These patients constituted the entire eligible cohort that met the inclusion and exclusion criteria during this 2-year period. The cohort consisted of 13 males and 24 females aged between 8 and 13 years (mean age: 10.78 ± 1.55 years). The spherical equivalent (SE) ranged from −1.50 D to −6.00 D, with a mean of −2.84 ± 1.11 D. Astigmatism was ≥ −1.25 D, with a mean of −0.69 ± 0.26 D. All participants had a best-corrected visual acuity (BCVA) of at least 0.00 logMAR in at least one eye.

Inclusion criteria were as follows: absence of significant refractive anomalies, no coexisting ocular diseases, no history of ocular trauma or surgery, and no systemic conditions that might interfere with contact lens fitting, including systemic allergies, autoimmune diseases, infectious disorders, or other systemic illnesses. Exclusion criteria included abnormal ocular findings such as moderate to severe dry eye, ocular hypertension, and markedly abnormal corneal curvature (either too flat or too steep).

This study was approved by the Ethics Committee of Ningde Municipal Hospital of Ningde Normal University (Number: 20200331) and conducted in accordance with the principles outlined in the Declaration of Helsinki. Written informed consent was obtained from all participants prior to enrollment, and clinical examinations were performed by experienced ophthalmologists.

### 2.2 Lens selection

Cerviño et al. (29) demonstrated that lens color affects glare perception, prompting the use of colorless MFSCLs and spectacles in our study. Additionally, since even minor lens contamination (e.g., fingerprints) exacerbates glare (30), all 37 spectacles were rigorously cleaned, and only new MFSCLs were utilized. This study employed aspheric continuous progressive Biothin MFSCLs made from Ocufilcon D silicone hydrogel [55% water content, Dk 19 × 10^−11^ (cm^2^/s) (mlO_2_/ml·mmHg), Dk/t 23.75 × 10^−9^ (cm/s) (mlO_2_/ml·mmHg)]. The lenses had an optical center thickness of 0.08 mm, a total diameter of 14.2 mm, 8.6 mm optical zone, 1.409 refractive index, >95.5% light transmission, and were daily disposable. Spectacles contained single-vision colorless lenses (1.67 refractive index) without visible scratches.

After enrolling the study subjects, a routine ophthalmologic examination was conducted by the same professional optometrist using a slit-lamp biomicroscope prior to lens dispensing. Based on the parameters of subjective refraction, PD, and monocular BCVA, the fit of the lenses was evaluated 15 min after insertion, focusing on centration, coverage, tightness/looseness, and subjective sensation. An ideal fit was defined as the lens being centrally positioned on the cornea when the subject was looking straight ahead, as observed through a slit lamp. The lens should fully cover the cornea, extending 0.5–2 mm beyond the corneal limbus, with the optical zone consistently covering the pupil area. The lens should exhibit 0.5–1.5 mm of movement during transient vision, and the subject should experience lasting comfort, stability, and clear vision. Glare assessment was conducted after confirming the fit to be ideal.

### 2.3 Visual function assessment

#### 2.3.1 Slit lamp biomicroscope

Before contact lens fitting and SDT assessment, all participants underwent a comprehensive anterior segment health evaluation, including slit-lamp examination of the cornea, conjunctiva, and tear film. This was to ensure ocular surface health was suitable for lens wear and to rule out any pathology that could interfere with visual quality assessments. Only participants with healthy anterior segment status were enrolled. Anterior segment health was assessed using a slit-lamp biomicroscope and the Efron Rating Scale (0–4), evaluating bulbar conjunctival congestion, corneal stromal edema, corneal punctate staining, giant papillary conjunctivitis, superior limbic keratoconjunctivitis, corneal infiltrates, and corneal ulcers (31).

#### 2.3.2 Comprehensive optometry

Cycloplegic refraction was followed by non-cycloplegic refraction the next day, culminating in comprehensive refraction to determine the final prescription. Refractive errors (myopia, hyperopia, and astigmatism) were measured with the Comprehensive Optometry system (Topcon, Japan), and BCVA was determined using the International Standard Logarithmic Visual Acuity Scale (ISLVS).

#### 2.3.3 Pupil diameter

Pupil diameter was measured using the NeurOptics VIP-200 Pupillometer after 15–20 min of dark adaptation. Three measurements were taken for each eye, and the average value was recorded to the nearest 0.01 mm.

#### 2.3.4 Corneal curvature

Corneal curvature was measured with the Medmont E300 Corneal Topographer, which provided detailed topographical data (32 rings, 15,120 data points) to calculate the mean CC.

### 2.4 Shape discrimination threshold test

#### 2.4.1 Apparatus

**Figure 1:**
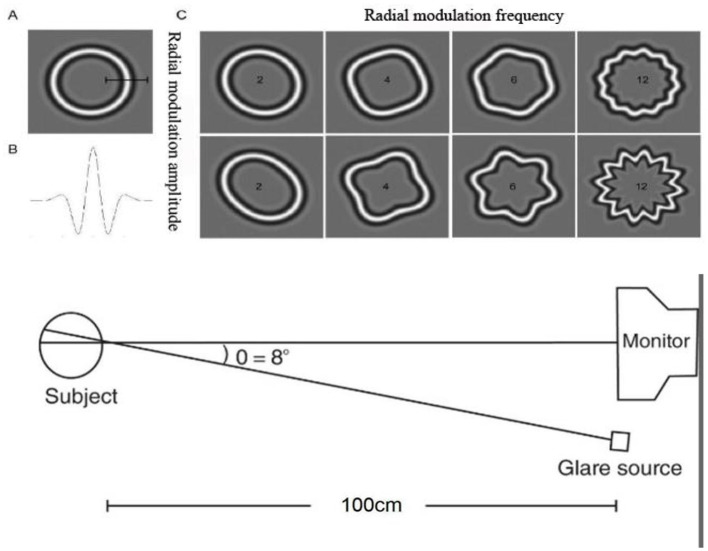
Schematic diagram of the RF patterns and SDT measurement platform. **(A)** standard circle, **(B)** radial modulation amplitude, **(C)** deformed circles.

The shape discrimination threshold (SDT) measurement platform was developed using MATLAB (Version 2016b, MathWorks Inc., Natick, Massachusetts, USA) and the Psychophysics Toolbox, as shown in Figure 1. This instrument demonstrated high reliability and repeatability (32). The measurement principle was grounded in a psychophysical approach and operated as follows. All stimuli were presented on an LED display (BL2710PT, BenQ) with a resolution of 2,560 × 1,440 pixels, and the mean luminance was set to 4–10 cd/m^2^, which was comparable with nighttime vision levels. The stimulus contrast was set at 20%, the radial frequency (RF) was 4, and the spatial frequency of the stimulus peaked at 3.0 cycles per degree, with a mean radius of 1.5°. Figure 1 illustrates the experimental layout, featuring a monitor positioned 1 m from the subject, with the source of glare located to the right of the monitor screen. The angle between the visual axes is relatively small (~8°), and the duration of the glare is 500 milliseconds, synchronized with the visual stimulus.

#### 2.4.2 Stimuli

The RF patterns were selected as visual stimulus (33). The underlying pattern was a modulated circumference, with the radial sinusoidal modulation radius described by the following equation:


$$\begin{array}{l} \text{r}\left(\text{θ}\right)=\text{r}0\left(1+\text{A}\sin\left(\text{wθ}+\text{φ}\right)\right) \end{array}$$

where r and θ are the polar coordinates of the pattern, *r*0 is the mean radius, A is the radial modulation amplitude (ranging from 0 to 1), *w* is the RF, and φ is the phase of the pattern. When the radial modulation amplitude (A) is set to 0, the visual stimulus appears as a standard circle. As A increases, the stimulus progressively distorts into a circle with varying degrees of distortion. Examples of RF patterns are shown in Figure 1. In each trial, a screen displaying a deformed circle and a standard circle was presented in random order. After multiple reversals at a specific stimulus level, the experiment concluded with a computer-generated average SDT, which served as an indicator of shape discrimination ability. A higher threshold value indicated poorer discrimination ability. The following equation was used


$$\begin{array}{l} \text{arc of sec}=\text{threshold}\times \text{R}0\times 3600 \end{array}$$

where arc of sec is measured in arc-seconds, threshold represents the SDT, denoting the smallest detectable deformation difference, and R0 represents the initial radius of the stimulus shape as it forms the viewing angle of the human eye.

#### 2.4.3 Procedures

For the glare test, subjects were fitted with MFSCLs and spectacles according to a computer-generated random sequence, and their visual acuity in the right eye was corrected to >0.00 logMAR. All participants underwent cycloplegic refraction followed by non-cycloplegic refraction the next day, culminating in comprehensive refraction to establish the final optical prescription. Spectacle prescriptions were optimized to achieve BCVA ≥ 0.00 logMAR in the right eye, verified through trial lenses before testing. All experiments were conducted in a dark environment, with the average room luminance set at 0.4 cd/m^2^. Initially, subjects underwent dark adaptation for 15 to 20 min. Subsequently, their sitting posture and head position were adjusted, along with the position of the jaw rest, to ensure proper alignment. The subjects were instructed to focus on the center of the screen directly in front of them, while their left eye was covered, and the test was performed on their right eye. The first measurement was initiated by pressing the measurement key under non-glare light conditions. Upon hearing a beep, standard and deformed circles were presented randomly and alternately, each for 500 ms, with a 250-ms interval between presentations. A 250-ms inter-stimulus interval better approximated the timing of rapid visual events, enhancing the task's ecological validity for capturing glare-induced interference within this critical temporal window. Furthermore, this parameter optimized efficiency while reducing observer fatigue. Subjects were instructed to identify which circle was deformed—the standard or the altered one—and to press either 1 or 2 on the keypad, corresponding to the order in which the circles appeared. The next set of patterns appeared with a beep after the subject made a choice, and the degree of deformation in that set was adjusted based on the subject's previous judgment. The staircase method of psychophysical measurement was employed, where two consecutive correct responses to a stimulus led to a decrease in stimulus intensity and one incorrect response led to an increase (34). This process was repeated to progressively approach the subject's SDT until they could no longer differentiate between a standard circle and a deformed circle, thereby determining the subject's SDT. After completing the initial measurements, the process was repeated two times and the average value was calculated to determine the final SDT under glare-free lighting conditions. Subjects were then instructed to focus on the center of the screen, avoiding the right side under glare illumination conditions. The measurements were repeated two times, and the average value was calculated as the final SDT under glare conditions. The interval between SDT assessments with MFSCLs and spectacles was 5–10 min, during which the subjects rested with their eyes closed. All measurements were completed within half an hour.

**Figure 2:**
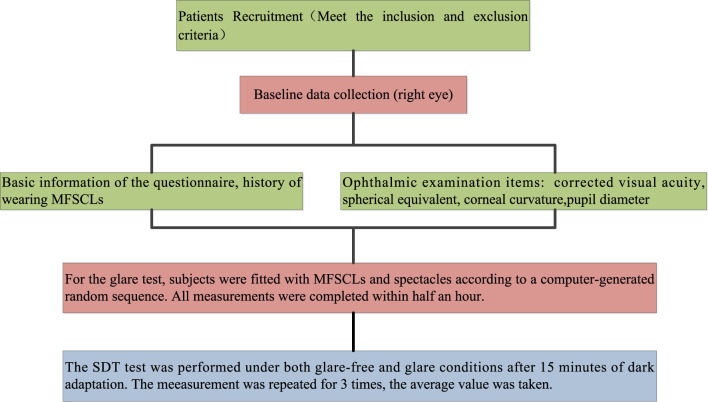
Flowchart of the experimental data collection process.

All subjects underwent a comprehensive ophthalmic examination conducted by the same professional optometrist. This examination included a slit-lamp biomicroscopy to assess lens fit and evaluate the health of the anterior segment of the eye. Additionally, the examination measured uncorrected visual acuity (UCVA), corrected visual acuity (CVA), CC, PD, SDT without glare (SDToff), and SDT with glare (SDTon). The data collection process is illustrated in Figure 2.

#### 2.4.4 Statistical analysis

Statistical analyses were conducted using SPSS version 26.0 (IBM Corp, Armonk, NY, USA). The Kolmogorov–Smirnov test was used to identify significant outliers and assess the conformity of the data to a normal distribution. Due to the non-normal distribution of most of the sample data, the assumption of normality could not be met. Therefore, non-parametric tests and correlation analyses were employed for each measurement. The Wilcoxon signed-rank test was used to compare the difference in SDT with and without glare. Additionally, the Spearman's correlation test was conducted to analyze the correlation between SDT and baseline information after wearing MFSCLs and spectacles. The mean and standard deviation (SD) of three repeated measurements were applied to determine descriptive statistics. The results were expressed as mean ± standard deviation, with differences considered statistically significant at a *P*-value of < 0.05.

## 3 Results

This study investigated the changes in SDT in children with myopia wearing MFSCLs and spectacles under both glare and non-glare conditions.

### 3.1 Baseline characteristics

Baseline characteristics of the 37 enrolled subjects are presented in Table 1. Effect size estimates (Cohen's d) were calculated for key comparisons to complement *P*-values. The glare-induced increase in SDT (SDTon vs. SDToff) showed large effect sizes in both lens groups (MFSCLs: *d* = 1.64; spectacles: *d* = 1.46).

**Table 1:** Baseline characteristics of the study subjects.

**Parameter**	**Mean ±SD**	**Range (min–max)**
Gender, *n* (%)	Male: 13 (35%)	N/A
	Female: 24 (65%)	N/A
Age (years)	10.78 ± 1.55	8 to 13
Spherical equivalent (D)	−2.84 ± 1.11	−1.5 to −6.0
Mean corneal curvature (D)	43.7 ± 1.17	41.63 to 46.5
Pupil diameter in a dark room (mm)	7.43 ± 0.47	6.27 to 8.45
Corrected visual acuity with MFSCLs (logMAR)	−0.10 ± 0.11	−0.08 to 0.00
Corrected visual acuity with spectacles (logMAR)	−0.06 ± 0.12	−0.08 to 0.00

Continuous variables are presented as mean ± standard deviation and range (minimum–maximum). Categorical variables marked N/A. VA, visual acuity; MFSCLs, multifocal soft contact lenses; logMAR, logarithm of the minimum angle of resolution.

### 3.2 Evaluation of MFSCL fit

After 37 study subjects wore the MFSCLs for 15 min, the fit was evaluated based on centration, coverage, and tightness/looseness. All lenses were found to be fitted without significant deviations from the ideal fit (as shown in Supplementary Table 1).

### 3.3 SDTs for MFSCLs vs. spectacles

**Figure 3:**
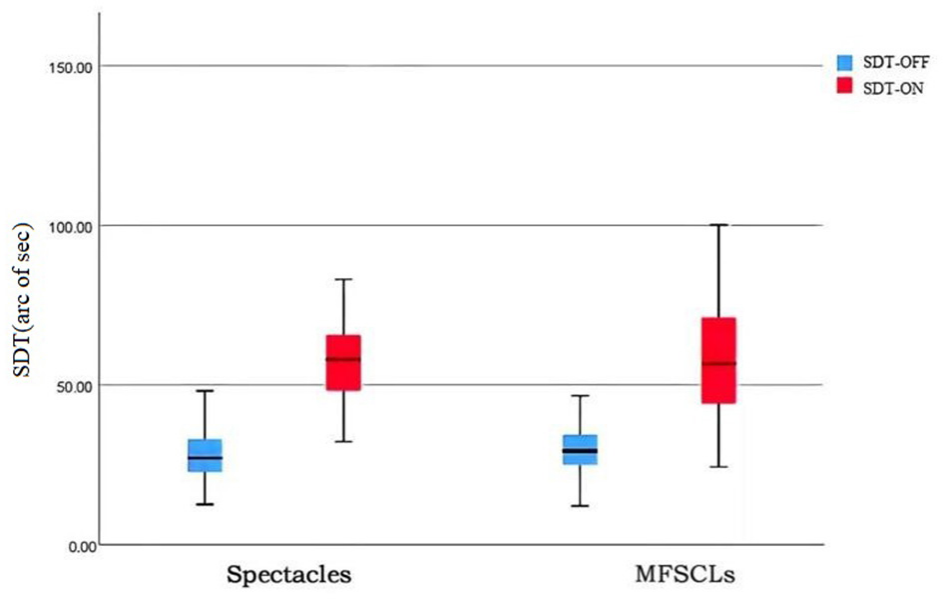
Comparison of SDTs between MFSCLs and spectacles with and without glare.

Shape discrimination thresholds with glare and without glare were analyzed using a non-parametric test (Wilcoxon signed-rank test) with paired samples. Without glare, the SDTs of the subjects' eyes were 30.86 ± 9.05 with MFSCLs and 28.18 ± 8.47 with spectacles, showing no statistically significant difference (*P* > 0.05; as shown in Table 2, Figure 3). With glare, the SDTs of the subjects' eyes were 59.56 ± 21.08 with MFSCLs and 59.85 ± 20.40 with spectacles, and the difference was not statistically significant (*p* > 0.05). The difference between SDToff and SDTon with and without glare was statistically significant (*P* < 0.001; as shown in Table 2, Figure 3).

**Table 2:** Comparison of SDToff, SDTon, and SDTdiff after wearing MFSCLs and spectacles (X ± S).

**Lens type**	**Number of samples**	**SDToff**	**SDTon**	**SDTdiff**	**P(SDTon vs. SDToff)**
MFSCLs	37	30.86 ± 9.05	59.56 ± 21.08	28.71 ± 17.52	< 0.05
Spectacles	37	28.18 ± 8.47	59.85 ± 20.40	31.68 ± 21.66	< 0.05
P		0.065	0.512	0.910	

SDToff is the SDT without glare, SDTon is the SDT with glare, and SDTdiff is the difference between SDTon and SDToff. Wilcoxon signed-rank test. The difference was statistically significant when P < 0.05.

### 3.4 Correlation analysis

**Figure 4:**
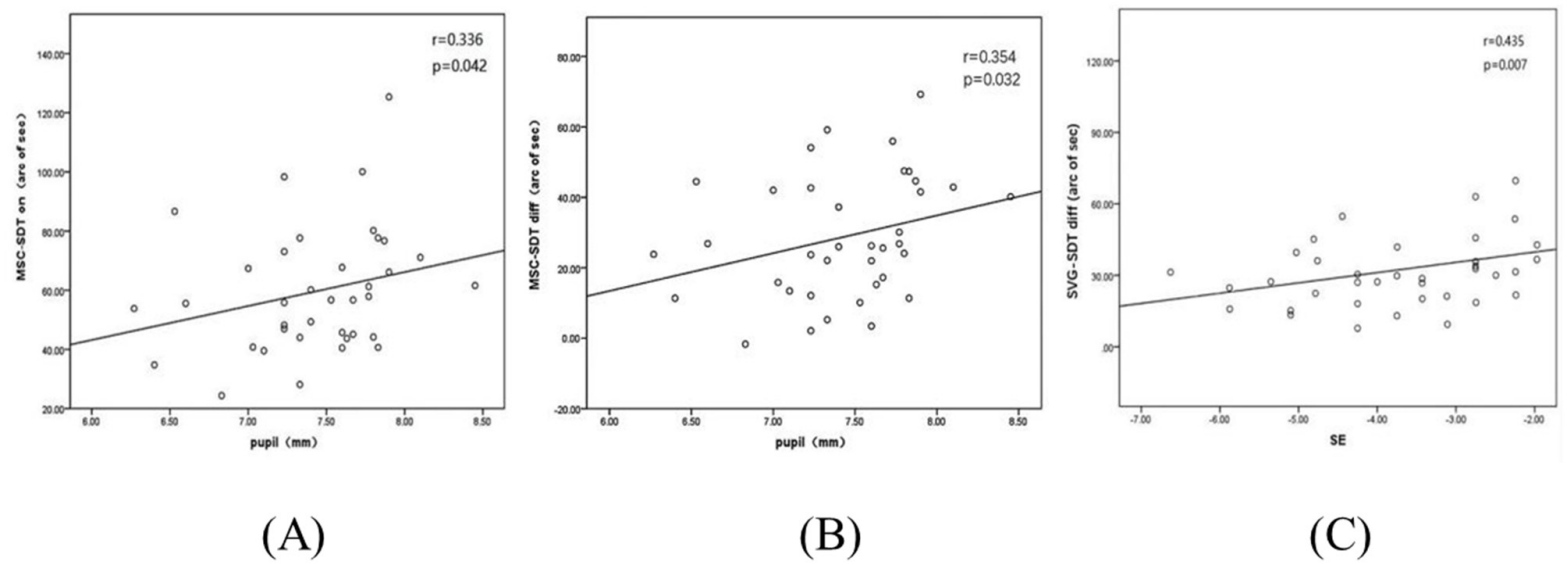
**(A)** SDTs of MFSCLs with glare correlate with pupil diameter measured in a darkroom. **(B)** Correlation between the difference in SDTs with and without glare and pupil diameter in a dark room with MFSCLs. **(C)** Correlation between the difference in SDTs with and without glare and spherical equivalent (SE) with spectacles.

Spearman's correlation analysis was conducted to explore the relationship between SDTs and the baseline parameters of the study subjects. A significant positive correlation was observed between the SDTs with glare and PD in a dark room when wearing MFSCLs (*r* = 0.336, *p* = 0.042), as shown in Figure 4A. Additionally, the difference in SDTs between glare and non-glare conditions was significantly correlated with PD in the dark room (*r* = 0.354, *p* = 0.032), as illustrated in Figure 4B. The difference in SDTs with glare and without glare was also significantly associated with the spherical equivalent after wearing spectacles (*r* = 0.435, *p* = 0.007), as presented in Figure 4C. There was no significant correlation between any of the other indicators, such as age, sex, and mean CC, as revealed in Table 3.

**Table 3:** Correlation between SDToff, SDTon, and baseline data after wearing MFSCLs and spectacles.

**Parameter**	**Age**		**Spherical equivalent**		**Pupil diameter in a dark room**		**Mean corneal curvature**	
	***r***	***p***	***r***	***p***	***r***	***p***	***r***	***p***
MSC-SDToff	0.086	0.612	−0.318	0.055	0.026	0.876	0.270	0.106
MSC-SDTon	−0.132	0.435	−1.91	0.259	0.336	< 0.05	0.252	0.133
MSC-SDTdiff	−0.097	0.569	−1.73	0.306	0.354	< 0.05	0.222	0.186
SVG-SDToff	0.009	0.956	−1.162	0.337	0.159	0.347	0.152	0.368
SVG-SDTon	−0.09	0.596	0.276	0.098	0.198	0.239	0.111	0.512
SVG-SDTdiff	−0.023	0.893	0.435	**< 0.05**	0.109	0.522	0.075	0.658

Spearman's correlation analysis. The correlation is significant when a p-value of < 0.05, and r is the correlation coefficient. MSC-SDToff is the SDT after wearing MFSCLs without glare. MSC-SDTon is the SDT after wearing MFSCLs with glare. MSC-SDTdiff is the variation of MSC-SDToff and MSC-SDTon. SVG-SDToff is the SDT after wearing spectacles without glare. SVG-SDTon is the SDT after wearing spectacles with glare. SVG-SDTdiff is the variation of SVG-SDToff and SVG-SDTon. The bold values indicates SVG-SDTdiff was associated with the SE (*P* < 0.05).

### 3.5 The health of the eye section

None of the study subjects had serious adverse events such as corneal epithelial pitting staining of grade 2 or higher, no corneal infiltrates, infections, or ulcers, and minor corneal epithelial pitting staining recovered well after prompt treatment, and none of the fittings were significantly off-set. The Efron rating scale suggested the presence of only minor changes in the anterior segment of the eye and the absence of any serious complications associated with the fitting of MFSCLs, indicating that the anterior segment of the eye did not show any lesions that could have caused an increase in the subject's glare, as revealed in Supplementary Table 2.

## 4 Discussion

The visual quality of MFSCLs as a method for preventing and controlling myopia in children has attracted considerable attention. Glare is a significant visual impairment that can negatively affect visual health and quality of life. In this study, the SDTs of 37 children with myopia were measured using psychophysical methods with glare and without glare conditions to evaluate the effect of MFSCLs. It was found that glare significantly increased the SDT and reduced shape discrimination ability. However, MFSCLs did not significantly increase the SDT compared with spectacles, suggesting that MFSCLs did not exacerbate disability glare.

Under glare conditions, the SDTs of the subjects were significantly higher, indicating that glare impaired shape discrimination ability. This may be attributed to the reduced image contrast and the partial masking effect caused by glare, which impacted the accuracy of subjects' judgments of figure contours. This phenomenon further supported the application of SDTs to quantitatively assess subjects' disability glare. There was no significant difference in SDTs between MFSCLs and spectacles, indicating that MFSCLs did not significantly increase disability glare. This result was corroborated by a study conducted by Gaurisankar et al. (35), which found no significant difference in glare between soft contact lenses and spectacles in the myopic group. van der Meulen et al. (36) measured stray light using the Oculus C-Quant and found no significant difference in stray light between wearing and removing soft contact lenses. However, a study by Nio et al. (37) found a significant increase in stray light following correction with soft contact lenses compared with other correction methods, such as spectacles. This may be related to factors such as the use of non-new soft contact lenses, inconsistent materials, wear and tear from inadequate lens care, or surface deposits. Wahl et al. (16) found significant differences in the disability glare induced by various designs of MFSCLs, while Łabuz et al. (38) attributed the substantial increase in stray light from MFSCLs to the lens material. The MFSCLs used in this study featured an aspheric continuous tapering design, which ensured a seamless change in refractive error and minimized light scattering, thereby providing high-quality vision for the subjects. Despite the effective control of disability glare by MFSCLs, some wearers in clinical settings still reported symptoms, such as visual fatigue and photophobia, which may be attributed to discomfort glare. Future research should further investigate the impact of MFSCLs on visual quality and explore effective strategies for controlling or avoiding glare.

Previous studies had indicated that wearing MFSCLs may result in ocular hypoxia, tear film instability, and dry eyes (39). Additionally, it may contribute to increased light scattering, visual blurring, higher-order aberrations, and deposits on the lens surface, all of which can exacerbate glare (40, 41). However, these studies measured glare exclusively through retinal image contrast. The current study included both glare-induced contrast degradation and image masking as influencing factors, and the study was conducted to identify shape with transient glare, providing a more realistic assessment of the effects of glare on visual functioning.

Significant correlations were observed between PD and SDTs in a dark environment with glare illumination after wearing MFSCLs. No such correlation was found under non-glare conditions. This may be related to the aspheric continuous progressive design of MFSCLs, which can be more prone to inducing myopic defocus and halos, especially in children with larger pupils (14). In the presence of glare, light was unevenly scattered through the refractive medium, producing stray light that reduces retinal image contrast and impairs shape discrimination ability. In the absence of glare, pupil size did not significantly impact retinal image contrast and had a minimal effect on disability glare. In addition, while shape discrimination ability decreased with increasing PD after wearing spectacles, this difference was not statistically significant. This lack of significance was likely due to minimal changes in stray light values, which were insufficient to cause substantial visual interference. Previous studies had suggested that higher-order aberrations in the human eye increased substantially with PD after wearing corneal contact lenses (42). This study was conducted in a simulated nighttime environment to assess SDTs and PDs. Therefore, in the presence of glare, pupil size may significantly impact visual quality for subjects wearing MFSCLs. However, there was limited research into the correlation between PD, glare, and other visual qualities in the context of MFSCLs. Further in-depth studies will be needed to clarify these relationships.

This study also analyzed the correlation between SDTs and the spherical equivalent. It was found that the difference in SDTs with and without glare after wearing spectacles was significantly correlated with the spherical equivalent. However, no statistically significant correlation was observed between SDTs and the spherical equivalent when using MFSCLs. Gaurisankar et al. (35) investigated the impact of various levels and modalities of refractive correction on glare. Their study found significant differences in glare measurements induced by negative lenses with different refractive indices, which supported this finding. We hypothesized that this observed discrepancy may be due to changes in the size of retinal light scatter spots in the subjects' eyes after wearing spectacles. The difference in spectacle magnification caused by negative lenses with varied refractive errors can lead to variations in the size of retinal images. The significant SDT-SE association observed with spectacles, but absent with MFSCLs, originated from fundamental optical differences in retinal image magnification (RIM). Spectacles induce SE-dependent minification: higher-minus lenses progressively reduce image size, systematically altering retinal light spread. This modifies perceived illuminance and veiling glare under testing conditions, explaining SDT-SE correlation. Conversely, MFSCLs' corneal placement minimizes SE-independent RIM changes, avoiding systematic light spread variations. Their secondary effects (e.g., contrast loss) lack SE correlation, accounting for non-significant SDT associations. Thus, SE-dependent scaling in spectacles (amplified by glare-sensitive light spread) drives this mechanistic divergence. Therefore, the spherical equivalent may not significantly affect the visual quality of subjects after wearing MFSCLs. However, there is a scarcity of research examining the correlation between spherical equivalent and visual quality factors, such as glare, following the application of MFSCLs. Thus, more in-depth studies will be necessary to clarify this issue.

The VA difference between wearing MFSCLs and spectacles (−0.10 vs. −0.06 logMAR, Table 1) reflected inherent differences between multifocal and single-vision optics. MFSCLs provide superior functional vision vs. spectacles by eliminating vertex distance effects and minimizing peripheral defocus. This enhances retinal image fidelity. The 0.04 logMAR VA advantage with MFSCLs reflects reduced optical aberrations from corneal apposition, particularly beneficial for children with large pupils (7.43 ± 0.47 mm). While statistically detectable, this difference is clinically insignificant (equivalent to < 2 ETDRS letters) and resides within identical functional ranges for both corrections (−0.08 to 0.00 logMAR, Table 1). Critically, it does not covary with SDT measures, as SDT assesses hyperacuity mechanisms distinct from high-contrast VA resolution. No significant correlation was found between visual acuity differences and changes in SDT results.

Despite these insights, the study has several limitations. The sample size was relatively modest, and only one design of MFSCLs was tested, limiting the generalizability of the findings. Additionally, while the glare simulation was controlled, real-world visual environments are more variable. Future research should include diverse lens designs and materials, larger participant cohorts, and combine both subjective and objective evaluations of glare. Longitudinal studies are also needed to assess whether prolonged MFSCL wear influences glare adaptation or other aspects of visual function in children.

## 5 Conclusion

This study demonstrated that children with myopia wearing MFSCLs exhibited SDTs comparable with those wearing spectacles, both under glare and non-glare conditions. These findings indicate that MFSCLs do not exacerbate disability under glare or compromise functional visual quality. Moreover, the SDT proves to be a sensitive and effective psychophysical measure for quantitatively evaluating visual performance under glare, offering valuable insights beyond conventional visual acuity assessments.

## Data Availability

The raw data supporting the conclusions of this article will be made available by the authors, without undue reservation.
