# Supplementary material for: Impact of multifocal soft contact lenses on the shape discrimination threshold under glare in myopic children
Source: Front Med (Lausanne). 2025 Aug 29;12:1635583. doi: 10.3389/fmed.2025.1635583 (PMC12425990; doi:10.3389/fmed.2025.1635583)
Supplement: Supplementary file 1 [file Table_1.docx]

**Supplementary Table 1**. Matching evaluation of subjects wearing MFSCLs.

|  | **0** | **1** | **2** | **3** | **4** |
| --- | --- | --- | --- | --- | --- |
| Coverage | Full Coverage  (37) | Corneoscleral  Exposure  (0) |  |  |  |
| Centering | Well  (37) | Slight Deviation  (0) | Severe Deviation  (0) |  |  |
| Tightness/Looseness | Well  (37) | Looseness was acceptable  (0) | Looseness was unacceptable  (0) | Tightness was acceptable  (0) | Tightness was  unacceptable  (0) |

Note: Grading criteria: 0=Ideal, 1-4=Suboptimsl, values in bracket are the number of subjects.

None of the study subjects had serious adverse events such as corneal epithelial pitting staining of grade 2 or higher, no corneal infiltrates, infections or ulcers, and minor corneal epithelial pitting staining recovered well after prompt treatment, and none of the fittings were significantly off-set. The Efron rating scale suggested the presence of only minor changes in the anterior segment of the eye and the absence of any serious complications associated with the fitting of MFSCLs, representing that the anterior segment of the eye did not show any lesions that could have caused an increase in the subject's glare as revealed in Supplementary Table 2.
