# Supplementary material for: Impact of multifocal soft contact lenses on the shape discrimination threshold under glare in myopic children
Source: Front Med (Lausanne). 2025 Aug 29;12:1635583. doi: 10.3389/fmed.2025.1635583 (PMC12425990; doi:10.3389/fmed.2025.1635583)
Supplement: Supplementary file 2 [file Table_2.docx]

**Supplementary Table 2**. Efron rating scale

| Complication | Average Value | Range |
| --- | --- | --- |
| Corneal punctate staining | 0.27 | 0-1 |
| Corneal stromal edema | 0 | 0 |
| Corneal infiltrates | 0 | 0 |
| Bulbar conjunctival congestion | 0.08 | 0-1 |
| Palpebral conjunctival abnormalities | 0.27 | 0-1 |
| Total score | 0.62 | 0-3 |

Note: The score from 0 (normal) to 4 (severe) indicated an increase in the severity of complications.
